# Conceptualizing Integration: A Framework for Analysis Applied to Neglected Tropical Disease Control Partnerships

**DOI:** 10.1371/journal.pntd.0000174

**Published:** 2008-04-30

**Authors:** Karen A. Grépin, Michael R. Reich

**Affiliations:** 1 Department of Health Policy, Harvard University, Cambridge, Massachusetts, United States of America; 2 Department of Population and International Health, Harvard School of Public Health, Boston, Massachusetts, United States of America; Swiss Tropical Institute, Switzerland

## Introduction

The timely and sustained delivery of effective health interventions to communities in developing countries is one of the greatest challenges in global health. Millions of the world's poorest citizens continue to be afflicted by bacterial, viral, and parasitic infections that have persisted, mainly in the tropics—the so-called neglected tropical diseases (NTDs)—despite the availability of safe and cost-effective interventions for the control and elimination of many of these diseases. Access to these interventions (or control tools) remains low and inadequate, particularly in sub-Saharan Africa [1],[2]. The NTDs, including onchocerciasis, schistosomiasis, lymphatic filariasis, trachoma, and soil-transmitted helminthiasis, have been shown to affect the poorest of the poor disproportionately. Addressing the NTDs, therefore, will be an essential element in poverty alleviation programs [3],[4].

A number of important international single-disease control partnerships have been developed over the last few decades [5]–[7]. To date, however, there has been little integration among these partnerships [3]. Integration refers to the creation of linkages among existing programs to improve the delivery of health interventions given existing commitments and resources. The presence of many common elements and general arguments about economies of scale provide strong reasons to believe that integration amongst partnerships can help improve both efficiency and effectiveness.

Interest in integration is currently at an all-time high, due in part to new funding for integration (the Bill & Melinda Gates Foundation has announced research grants to investigate integration of NTDs, and the United States Agency for International Development [USAID] has awarded operational grants to scale-up integrated NTD control programs), the creation of the Global Network for Neglected Tropical Diseases Control (GNNTDC; http://gnntdc.sabin.org/), and high-level political commitment to address these scourges [8]. In addition, reports of successful national control programs for single diseases supported by these partnerships (such as trachoma control in Morocco and lymphatic filariasis control in Egypt) bolster the case that integration be prioritized in affected countries.

While there has been significant discussion about the integration of single-disease partnerships [9]–[11] and the potential usefulness of such approaches in helping to tackle the burden of NTDs, there is limited experience in implementing integration and even less experience in conducting systematic analysis of these experiences. Recently, a number of articles have discussed potential challenges and opportunities, and have estimated potential benefits, including cost savings [12]–[14].

The lack of a common understanding of integration for disease control programs may be a significant impediment towards implementing integration, despite significant interest in the topic. This article presents a conceptual framework to help guide the discussion about integration of NTD control partnerships. It then provides specific examples of potential opportunities and actual cases of integration of NTDs, and places these examples within the conceptual framework. The main purpose of this article is to provide a tool for thinking about integration—to aid the development, implementation, and evaluation of future efforts at integrating NTD control programs. This framework could also be used for assessing other forms of integration among service-oriented programs. This article does not provide lessons from ongoing NTD integration efforts, because the existing attempts are at too early a stage to generate results.

## Conceptualizing Integration

Integration has been interpreted to mean different things to different organizations and individuals. In fact, many different options exist for integration. To understand the differences among these options, it is important to define with some precision the dimensions along which integration can occur. The following framework can be used to conceptualize the options based on differences in domain, level, and degree of integration.

### Domain: What Is Being Integrated?

Building upon the framework for integration of human services developed by Agranoff and Pattakos, integration can occur within a number of different domains [15]. Distinguishing among these different domains helps to answer the question, *what is being integrated*? The following provides a description of each domain using health examples:

- **Activity domain:** Joining core activities of separate programs. Activity integration could involve joint distributions, multi-disease evaluations, or joint training sessions for community distributors.
- **Policy domain:** Joining the policy functions of separate programs, such as advocacy, needs and priority assessment, technical and financial guideline development, and programming and coordination activities. Policy integration could include the development of multi-disease indicators and harmonized incentive structures for community distributors.
- **Organizational structure domain:** Merging separate programs into a common structure or forming a new organization. Organizational integration could involve the formation of a new partnership for community-based distribution or the consolidation of one disease program into another where one disease program has clear comparative advantages over another.

### Level: Where Is Integration Occurring?

Integration can occur at different levels in a health system. The costs and benefits of integration will vary depending on the particular level targeted for integration. Distinguishing among the levels helps to answer the question, *where is integration occurring*? It is important to note that while integration can occur at any of these levels, integration at one level will likely have implications at other levels. The following provides an overview of three levels and examples of integration that could occur at each level:

- **Global:** Integration among the international partnerships and other international health organizations involved in the financing, planning, and implementing of disease-specific programs. For example, the major public–private partnerships could work collaboratively on the development of joint indicators for multi-disease program evaluations (i.e., in the policy domain).
- **National/regional:** Integration among national or regional disease-specific programs, various divisions within the Ministry of Health (MoH), other relevant public sector offices, non-governmental organizations (NGOs), and other national or regional partners. For example, the national program coordinators could coordinate their activities at the national level in order to guide the activities at the regional, district, and community levels (i.e., in the activity domain).
- **Local (district/village/community):** Integration among the implementers, including MoH employees, NGOs, community volunteers, and relevant community-based partners. For example, districts could consolidate the various training sessions for community distributors into a single training session (i.e., in the activity domain).

### Degree: How Is Integration Occurring?

Finally, for a given domain and level, the degree to which programs actually implement integration can also vary. Distinguishing among these different degrees helps answer the question, *how is integration occurring*? The following provides an overview of the different degrees over which integration could occur:

- **Coordination:** Communication and information exchange among distinct programs for the purpose of simplifying the implementation of the respective programs. For example, programs could work together at the national level to develop an annual plan for implementation (i.e., in the activity domain and at the national level).
- **Collaboration:** Increased cooperation among disease-specific programs, which, in addition to increased coordination, could include the sharing of resources or personnel. For example, multiple programs can join together to purchase vehicles and other equipment that could then be used by all of the programs (i.e., in the activity domain and at the national and regional levels).
- **Consolidation:** Implementation of a portion or an entire program by another program. Consolidation implies the replacement of either a portion or the entire program by a new effort or entity. For example, instead of conducting multiple single-disease training sessions for district-level health workers, regional-level health workers could instead offer a single once-a-year training session for multiple-disease programs (i.e., in the activity domain and at the implementation level).

## Examples of Integration for NTD Control Programs

To illustrate the above framework, we have taken real-world examples of and potential opportunities for integration in NTD control programs and have categorized them in Table 1 by level (global, national/regional, and local) and domain (activity, policy, and organizational), using the framework. For each example, the text in Table 1 describes the degree of integration.

**Table 1:** Examples of Integration for NTD Control Programs, Organized by Level and Domain.

		*LEVEL*		
		Global	National/Regional	Local
*DOMAIN*	**Activity**	Gates Foundation–funded operational research projects (collaboration)	Coordination of treatment distributions at national level	Joint training sessions for local distributors (collaboration)
	**Policy**	Coordinated guidelines for co-administration of treatments	APOC technical support to countries (coordination)	Harmonized incentives for local distributors (coordination)
	**Organizational**	The Global Network for Neglected Tropical Diseases Control (collaboration)	Multi-disease surveillance (consolidation)	Multi-disease drug distributors (consolidation)

Note: The text describing each example indicates the degree of integration (coordination, collaboration, and consolidation).

### Global/Activity:

**Gates Foundation–funded operational research projects (collaboration):** In the fall of 2006, the Bill & Melinda Gates Foundation awarded approximately US$46.7 million in research grants to multi-disease partnerships to investigate the costs and benefits of integrated approaches for NTDs [16]. Multiple organizations are involved in these grants, including the International Trachoma Initiative, the Schistosomiasis Control Initiative, the Centers for Disease Control and Prevention, Emory University, and the Carter Center. Research for these projects will take place in numerous countries and cover numerous diseases. Additional collaborative efforts include attempts to standardize costing methodologies across all of these Gates-funded projects.

### National-Regional/Activity:

**Coordination of distributions at national level (coordination):** In many countries, the timing and sequencing of drug distributions are coordinated at the national level and built into joint national operational plans. By coordinating these distributions at the national level, resources can be deployed more efficiently at the national and district levels towards these programs and additional opportunities for integration can be identified.

### Local/Activity:

**Joint training sessions (collaboration):** District health centers in certain communities conduct joint training session for community distributors and other health workers. Since many of the training sessions are similar in nature, training sessions can be shorter, and health workers and community distributors need only travel once to receive their complete training package.

### Global/Policy:

**Coordinated guidelines for co-administration (coordination):** The World Health Organization (WHO) recently completed guidelines for countries on integrated chemotherapies for helminthic infections [17]. These guidelines summarize the academic literature of the safety of such practices and provide suggested guidelines for the development of national guidelines for integrated control.

### National-Regional/Policy:

**APOC technical support to countries (coordination):** The African Programme for Onchocerciasis Control (APOC) has recently expanded its mandate to be able to provide assistance to national governments for the development of national policies on integrated NTD control that include onchocerciasis [18]. APOC is the longest running single-disease control program and has developed extensive expertise and knowledge in all aspects of disease control. By leveraging their expertise and contacts, countries may be able to more quickly develop new integrated disease control programs.

### Local/Policy:

**Harmonized incentives (coordination):** A key challenge identified by many of the single-disease control programs is the lack of standardized remuneration packages, which potentially creates harmful incentives for the community distributors. Villages could establish uniform compensation guidelines for community distributors.

### Global/Organizational:

**GNNTDC (collaboration):** In 2006, the GNNTDC was formed to coordinate advocacy and information dissemination efforts for integrated NTD control at the international level. The goal is to help link NTD control efforts with those that have been more broadly aimed at poverty reduction.

### National-Regional/Organizational:

**Multi-disease surveillance (consolidation):** In some countries, a single coordinator has been put in charge of national surveillance for multiple diseases. It may be possible for surveillance officers to collect samples for more than one disease during their visits to the infected areas, thereby saving time and other resources.

### Local/Organizational:

**Multi-disease drug distributors (consolidation):** In many villages, the same individual is made responsible for the distribution of more than one disease control program. If the same individual is made responsible for multi-disease distributions, it could reduce the amount of time these people need to devote to notifying their constituents, attending trainings, and implementing the distributions. It may also lead to important productivity gains since there is a learning curve associated with each of these programs.

## Conclusions

A common understanding of the concept of integration can help guide future discussions about the opportunities and challenges for integration among NTD control programs. The framework presented in this article provides a tool for clarifying the different domains, levels, and degrees of integration. This framework has already been used in two situations: to assist in the development of new integration strategies for APOC, and to guide the development of strategies to integrate trachoma and lymphatic filariasis control programs; in both instances, the framework was helpful.

The integration of NTD control programs offers the potential for improving the delivery of NTD control programs in resource-poor regions such as sub-Saharan Africa, where millions remain at risk of these diseases. We have provided some real-world examples of integrated NTD control activities and have demonstrated how these efforts can be categorized using the framework. There is a need to accelerate the implementation of integrated NTD control activities, but they also must be evaluated in a systematic manner. We believe this framework can help understand how to best create linkages among these programs and how to deliver much needed services to affected communities in more efficient and effective ways.
